# Association Between Bioelectrical Impedance-Derived Phase Angle and Functional Status in Post-Stroke Patients: A Retrospective Analysis

**DOI:** 10.3390/medicina62020357

**Published:** 2026-02-11

**Authors:** Soyeong Kim, Byeonggeun Kim, Seju Park

**Affiliations:** 1 Department of Research Affairs, Gwangju 365 Rehabilitation Hospital, Gwangju 62232, Republic of Korea; belleyou11@naver.com; 2 Regional Health & Medical Center for Persons with Disabilities, Chonnam National University Hospital, Gwangju 61469, Republic of Korea; qudrms_92@naver.com; 3 Department of Health and Safety Management, Songwon University, Gwangju 61756, Republic of Korea

**Keywords:** stroke, phase angle, function, convalescent rehabilitation

## Abstract

*Background and Objectives*: Phase angle (PhA) derived from bioelectrical impedance analysis reflects muscle quality and cellular integrity and has been associated with functional outcomes after stroke. However, its relationship with functional status at hospital admission and potential sex-based differences remains unclear. This study investigated the association between PhA and functional status at admission in post-stroke patients undergoing convalescent rehabilitation. *Materials and Methods*: This retrospective study included 250 post-stroke patients admitted to a convalescent rehabilitation hospital. PhA was measured at admission and classified into high and low groups using sex-specific cutoffs. Functional status was assessed using the Berg Balance Scale (BBS), Functional Ambulation Category (FAC), and the Korean version of the Modified Barthel Index (K-MBI). Sex-stratified logistic regression analyses were conducted to examine associations between functional variables and low PhA. *Results*: Patients with low PhA showed significantly poorer balance, ambulation, and activities of daily living (ADL) than those with high PhA. In men, low balance (BBS < 41) and low ambulation ability (FAC < 3) were independently associated with low PhA. In women, low ADL performance (K-MBI < 75) was independently associated with low PhA, while balance and ambulation were not. *Conclusions*: PhA was significantly associated with functional status at admission in post-stroke patients, with distinct sex-specific patterns. PhA may serve as a simple and non-invasive adjunct indicator of functional vulnerability when interpreted with consideration of sex differences.

## 1. Introduction

Stroke is an acute neurological deficit caused by cerebral infarction or hemorrhage and is one of the leading causes of mortality and disability worldwide [1,2]. After stroke, patients experience a wide range of neurological and functional impairments depending on the location and extent of the lesion, including muscle weakness, impaired motor control, reduced balance, gait dysfunction, decreased ability to perform activities of daily living (ADL), and deficits in cognitive and language function [1,3,4]. These functional impairments often persist beyond the acute phase into the convalescent stage, and the patient’s condition at the time of hospital admission is known to be a key determinant of subsequent functional recovery during rehabilitation [5,6]. Previous studies have reported that lower-limb muscle strength and balance ability in post-stroke patients are closely associated with ambulation capacity [7,8], while impairments in upper-limb function are major limiting factors for independence in ADL [9]. In addition, fatigue and poor nutritional status reduce participation in rehabilitation and act as factors that delay functional recovery [10,11].

In recent studies, body composition indices have been reported as important factors for predicting not only the overall health status of post-stroke patients but also subsequent functional recovery and prognosis [12,13]. In the past, the application of bioelectrical impedance analysis (BIA) in stroke patients was limited due to hemiparesis and restrictions in standing posture and handgrip function. Consequently, studies evaluating changes in body composition—particularly muscle mass—following rehabilitation interventions such as resistance training were limited [14]. However, recent advances in BIA measurement techniques, including methods applicable in the supine position or in patients with impaired handgrip function, have expanded the feasibility of body composition assessment in stroke populations. Accordingly, since 2020, the number of studies examining body composition in stroke patients has increased, and analyses of changes in body composition following rehabilitation interventions have been increasingly reported [15]. Within this context, phase angle (PhA) derived from BIA has gained attention as an indicator reflecting the physical condition of post-stroke patients [15,16]. PhA is considered an indirect marker of cellular mass and the balance of body water distribution and is interpreted as being more closely related to the structural and qualitative characteristics of muscle tissue rather than muscle quantity alone [17,18,19].

In previous studies, body composition characteristics in patients with stroke—including muscle mass, fat mass, and fat distribution—have been shown to differ according to sex and age, and these differences have been associated with functional recovery as well as nutritional and hydration status during rehabilitation [20,21]. Furthermore, sex-based differences in independence in activities of daily living and overall functional recovery after stroke have also been reported [22]. These findings suggest that sex is an important biological factor that should be considered when interpreting body composition–related indicators and their associations with functional status in post-stroke populations.

Several previous studies involving post-stroke patients have reported that PhA measured at hospital admission is associated with ADL at discharge, highlighting its potential predictive value [23,24,25]. However, these studies have primarily focused on the role of PhA in predicting post-discharge functional recovery or prognosis, and the extent to which PhA reflects patients’ functional status at the time of admission has not been sufficiently examined. In addition, prior research has been limited by the use of a single functional domain, such as ADL, or by the lack of a comprehensive analysis encompassing key functional domains including balance and ambulation. Therefore, there is a need to comprehensively evaluate the concurrent association between PhA and functional status at admission across multiple functional domains, including balance, gait, and ADL.

Therefore, this study aimed to examine the association between bioelectrical impedance–derived PhA measured at admission to a convalescent rehabilitation hospital and the functional status of post-stroke patients. Specifically, we investigated the relationships between PhA and balance ability, ambulation function, and ADL at the time of admission to determine whether PhA reflects patients’ initial functional status in the early stage of convalescent rehabilitation.

## 2. Materials and Methods

### 2.1. Study Design and Data Source

This study was a retrospective observational study conducted among post-stroke patients admitted to G Rehabilitation Hospital between 1 July 2022, and 30 June 2024. Electronic medical records routinely collected during clinical care were used to analyze clinical information at admission, body composition measurements, and functional assessment data. Prior to study initiation, ethical approval was obtained from the Ministry of Health and Welfare–designated Public Institutional Review Board (No. P01-202511-01-042). Given the retrospective nature of the study and the use of de-identified data, the requirement for informed consent was waived. All data were anonymized in accordance with ethical guidelines, and no personally identifiable information was included in the analysis.

### 2.2. Study Participants

The study population comprised patients admitted to G Rehabilitation Hospital between 1 July 2022, and 30 June 2024, who were diagnosed with stroke-related hemiparesis confirmed by magnetic resonance imaging or computed tomography. Inclusion criteria were as follows: admission to a convalescent rehabilitation hospital within 3 months after stroke onset, a hospital stay of at least 3 months, and the availability of relevant clinical information and functional assessment data related to stroke in the electronic medical records. In Korea, patients with stroke are eligible for intensive convalescent rehabilitation services in designated rehabilitation hospitals only when admitted within 3 months after stroke onset, according to the national rehabilitation medical system. This criterion was therefore applied to ensure that the study population represented patients in the convalescent stage receiving standardized, high-intensity rehabilitation care. The criterion of a minimum hospital stay of 3 months was applied to ensure the availability of complete and standardized functional assessment data at admission and during the early phase of convalescent rehabilitation.

**Figure 1:**
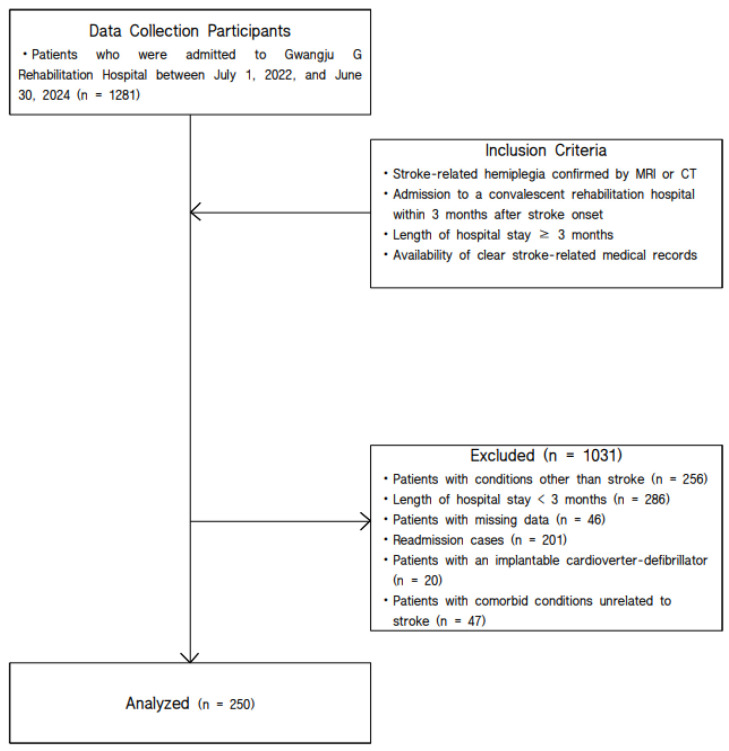
Flow diagram of participant selection.

Of the initial 1281 patients screened, those admitted for conditions other than stroke, those with a hospital stay of less than 3 months, patients with missing essential data, cases of readmission, patients with implanted pacemakers that could affect body composition measurements, and those with severe comorbid conditions unrelated to stroke were excluded. After applying these exclusion criteria, a total of 250 patients were included in the final analysis. The participant selection process is illustrated in Figure 1.

### 2.3. Subsection

#### 2.3.1. Body Composition Measurement

Body composition was assessed at the time of hospital admission using BIA performed as part of routine clinical care with a BIA device (BWA 2.0, InBody, Seoul, Republic of Korea). All measurements were conducted by trained clinical staff in accordance with the manufacturer’s guidelines. Depending on the participant’s physical condition, assessments were performed in appropriate positions as recommended by the device. Previous studies using the same BIA device have reported small differences in phase angle values across different measurement positions (standing, sitting, and supine); however, these differences were considered minimal and the measurements were deemed interchangeable [26]. The BIA assessment provided basic anthropometric data, including height and body weight, as well as skeletal muscle index and PhA. The BIA assessment provided basic anthropometric data, including height and body weight, as well as skeletal muscle index and PhA. Among the body composition indices, PhA was selected as the primary variable of interest in this study. PhA was dichotomized using sex-specific cutoff values reported in previous studies conducted in acute stroke populations, with cutoff points of 4.62° for females and 5.62° for males; values equal to or above the cutoff were classified as the high PhA group, whereas values below the cutoff were classified as the low PhA group [27].

Sarcopenia was diagnosed based on the Asian Working Group for Sarcopenia criteria by integrating skeletal muscle index and handgrip strength assessments [28], and the presence or absence of sarcopenia was treated as a categorical variable. All body composition and sarcopenia-related data were obtained from measurements recorded at admission in the electronic medical records.

#### 2.3.2. Functional Assessments

Functional status was evaluated at the time of hospital admission using standardized assessment tools routinely applied in clinical practice. Balance ability was assessed using the Berg Balance Scale (BBS) [29], while ambulation function was evaluated using the Functional Ambulation Category (FAC), which classifies the level of assistance required during walking [30]. ADL were measured using the Korean version of the Modified Barthel Index (K-MBI) [31]. When patients were unable to perform specific test items, scores were assigned according to the standardized scoring rules of each assessment tool, reflecting the lowest functional level.

For statistical analysis, each functional assessment was dichotomized according to predefined cutoff values. The BBS was categorized as ≥41 versus ≤40, the FAC as ≥3 versus ≤2, and the K-MBI as ≥75 versus ≤74, with higher scores indicating better functional status. Based on these classifications, functional status at admission was defined as categorical variables representing high and low functional levels.

### 2.4. Statistical Analysis

Descriptive statistics were used to summarize the general characteristics and body composition indices of the study participants. Continuous variables are presented as means and standard deviations, and categorical variables are presented as frequencies and percentages. Differences in general characteristics according to PhA level were analyzed using appropriate statistical methods based on the distribution of each variable. Continuous variables were compared using independent *t*-tests or Mann–Whitney U tests, depending on the results of normality testing, while categorical variables were compared using chi-square tests.

Logistic regression analyses were performed to evaluate the associations between PhA and functional indicators. Univariable logistic regression analyses were first conducted to examine crude associations between each functional indicator and low PhA. Subsequently, multivariable logistic regression analyses were performed to examine the independent associations between functional variables and low PhA. Because the primary aim was to evaluate whether PhA reflects functional status across different functional domains, the multivariable models included functional indicators only (BBS, FAC, and K-MBI) entered simultaneously, without adjustment for general clinical characteristics. This approach was chosen to avoid overadjustment and to focus on the direct relationships between functional performance and PhA.

All statistical analyses were conducted using IBM SPSS Statistics (version 27.0; IBM Corp., Armonk, NY, USA), and the level of statistical significance was set at *p* < 0.05.

## 3. Results

### 3.1. General Characteristics of the Participants

A total of 250 post-stroke patients were included in this study, comprising 119 males and 131 females. Participants were stratified by sex and subsequently classified into high and low PhA groups according to sex-specific PhA cutoff values (Table 1).

Among male participants, there were no significant differences between the high and low PhA groups in stroke etiology or affected side. In contrast, the prevalence of sarcopenia was significantly higher in the low PhA group than in the high PhA group (*p* = 0.001). Age was significantly higher in the low PhA group compared with the high PhA group (*p* < 0.001), while body weight (*p* = 0.005) and body mass index (*p* = 0.002) were significantly lower in the low PhA group. Height did not differ significantly between the two groups.

Similarly, among female participants, no significant differences were observed between the high and low PhA groups with respect to stroke etiology, affected side, height, or body weight. However, the prevalence of sarcopenia was significantly higher in the low PhA group than in the high PhA group (*p* < 0.001), and age was also significantly higher in the low PhA group (*p* < 0.001). Body mass index did not differ significantly between the two groups.

### 3.2. Comparison of Functional Outcomes According to Phase Angle Groups

When functional status was compared according to PhA level, both male and female patients in the low PhA group exhibited significantly poorer balance, ambulation, and ADL compared with those in the high PhA group (Table 2).

Among male participants, the BBS score was significantly lower in the low PhA group than in the high PhA group (*p* = 0.002). The FAC score was also significantly lower in the low PhA group compared with the high PhA group (*p* = 0.002). Similarly, the K-MBI score was significantly lower in the low PhA group than in the high PhA group (*p* = 0.001).

A similar pattern was observed among female participants. The BBS score was significantly lower in the low PhA group than in the high PhA group (*p* = 0.002). The FAC score was also significantly lower in the low PhA group compared with the high PhA group (*p* = 0.040). In addition, the K-MBI score was significantly lower in the low PhA group than in the high PhA group (*p* = 0.002).

### 3.3. Associations Between Phase Angle and Functional Variables

Sex-stratified logistic regression analyses were performed to examine the associations between PhA and functional variables, with low PhA set as the dependent variable (Table 3).

Among male participants, lower balance ability (BBS < 41) and reduced ambulation function (FAC < 3) were independently associated with low PhA (*p* < 0.05). These associations remained significant in multivariable models including all functional indicators, whereas ADL dependency (K-MBI < 75) was not significantly associated with low PhA.

In contrast, among female participants, ADL dependency (K-MBI < 75) was the only functional variable independently associated with low PhA (*p* < 0.05). Balance and ambulation measures were not significantly associated with low PhA in either univariable or multivariable analyses.

Overall, these findings indicate distinct sex-specific patterns in the associations between PhA and functional domains, with PhA reflecting balance and ambulation function in males and ADL performance in females.

## 4. Discussion

This study examined the association between BIA–derived phase angle and functional status at admission among post-stroke patients admitted to a convalescent rehabilitation hospital, with analyses stratified by sex. The results demonstrated that low PhA was significantly associated with functional impairment, and the patterns of these associations differed between males and females.

PhA reflects cellular membrane integrity and the distribution of intra- and extracellular body water and is considered a more comprehensive indicator of muscle quality and overall physiological status than quantitative measures such as muscle mass alone [32,33]. Previous studies have consistently reported that PhA is closely associated with muscle strength, physical function, and clinical prognosis [34,35]. In the present study, patients with low PhA demonstrated poorer balance, ambulation, and ADL performance, which is consistent with prior evidence suggesting that PhA reflects the physiological basis underlying neuromuscular activation and functional performance.

Notably, the associations between PhA and functional indicators differed by sex. In females, low PhA was independently associated with poorer ADL performance, whereas in males, low PhA was significantly associated with impairments in balance and ambulation. These sex-specific differences may be explained, at least in part, by variations in body composition, functional strategies, and the functional domains most susceptible to decline during recovery between males and females [36,37]. In general, females tend to have lower absolute muscle mass and may rely more on compensatory strategies during daily activities, which could lead to earlier manifestation of functional decline in the ADL domain [38]. In contrast, in men, mobility-related functions—particularly balance control and ambulation—may serve as key indicators reflecting overall functional status [39].

Beyond these functional explanations, additional physiological factors may also contribute to the observed sex-specific patterns. Differences in baseline muscle quality, muscle fiber composition, and neuromuscular efficiency between males and females may influence how PhA relates to specific functional domains. In addition, hormonal factors and sex-related differences in age-related muscle decline could modulate the relationship between cellular health, as reflected by PhA, and functional performance.

However, both the functional and physiological interpretations described above should be considered within the context of the study design. PhA represents an integrative indicator of body composition and cellular health, and its associations with functional outcomes may differ according to the functional domain being assessed. The observed sex-specific patterns may therefore reflect differences in how PhA relates to balance, ambulation, and ADL performance rather than a single underlying mechanism. In addition, stroke-related factors such as stroke severity, age distribution, or the involvement of dominant limbs, which were not explicitly examined in this study, may also contribute to the observed sex-specific associations. Although the study population covered a typical age range for patients admitted to convalescent rehabilitation after stroke, age-related heterogeneity may have influenced functional presentation and should be considered when interpreting the findings.

Meanwhile, although group comparisons showed that balance, ambulation, and ADL were consistently poorer in the low PhA group in both men and women, the functional domains significantly associated with PhA differed by sex in multivariable analyses. This finding suggests that the relationship between PhA and functional status is not a simple one-to-one association but rather reflects the combined influence of multiple factors, including age, body composition, and the characteristics of specific functional domains [33,40]. In other words, while PhA may be broadly associated with functional decline across multiple domains, after adjustment for other clinical factors, only the most representative functional domains may remain significantly associated with PhA in a sex-specific manner.

These findings indicate that PhA should be interpreted as an integrative indicator reflecting different patterns of clinical vulnerability depending on sex and functional domain, rather than as a marker of a single functional outcome [19,41]. Accordingly, PhA may be more appropriately understood as an indirect indicator of overall physical condition and risk of functional decline, rather than as a direct surrogate for a specific level of functional performance.

At admission to a convalescent rehabilitation hospital, functional status is typically evaluated through a combination of standardized functional assessments and clinical judgment by a multidisciplinary rehabilitation team. In this clinical context, despite its integrative nature, PhA may be most appropriately applied as a complementary indicator that provides additional insight into patients’ underlying physiological condition. Because PhA can be obtained rapidly and noninvasively as part of routine body composition assessment, it may support the interpretation of functional test results by helping to identify patients who appear functionally similar but differ in their physiological vulnerability. In this way, PhA may contribute to a more comprehensive understanding of patient status at admission, rather than replacing established functional assessments.

From a clinical perspective, the findings of this study suggest that PhA may serve as a useful indicator for the early identification of functional vulnerability in patients with post-stroke undergoing convalescent rehabilitation. Specifically, PhA may help identify the risk of decline in ADL performance in females and the risk of impairments in balance and ambulation in males. Interpreting PhA with consideration of these sex-specific patterns may contribute to the establishment of rehabilitation goals and the development of individualized intervention strategies.

This study has several limitations. First, due to its retrospective cross-sectional design, causal relationships between PhA and functional impairment could not be established. Second, as the data were derived from a single institution and restricted to patients with a hospital stay of at least three months, the findings may be subject to limited generalizability and potential selection bias. Third, the sex-specific PhA cutoff values were derived from studies conducted in acute stroke populations; therefore, their direct applicability to convalescent or subacute stroke patients may be limited. Fourth, although multivariable analyses focused on functional indicators to avoid overadjustment and to emphasize functional relationships, residual confounding by unmeasured clinical characteristics cannot be excluded. In addition, the dichotomization of PhA and functional measures may have resulted in loss of information and reduced statistical power, and the sex-stratified multivariable models may be subject to potential overfitting due to the limited number of events within each subgroup. Finally, because only admission data were analyzed, longitudinal changes in PhA and their relationship with functional recovery could not be evaluated. Future longitudinal studies are needed to clarify the prognostic role of PhA during the rehabilitation process, to explore the underlying cellular and physiological mechanisms linking PhA to post-stroke functional impairment, and to investigate long-term changes in functional status over months and years following stroke.

## 5. Conclusions

This study demonstrated that BIA–derived PhA is significantly associated with initial functional status in post-stroke patients admitted to a convalescent rehabilitation hospital, and that its clinical implications may differ by sex. Although low PhA was associated with overall functional impairment, multivariable analyses revealed distinct patterns of independent associations with specific functional domains in males and females.

These findings suggest that PhA should be interpreted not as a direct surrogate for a single functional outcome, but as an integrative physiological indicator reflecting different patterns of clinical vulnerability according to sex and functional domain. As a simple and non-invasive measure, PhA may have potential utility as an adjunct indicator for assessing functional status and estimating the risk of functional decline and prognosis in post-stroke patients when interpreted with consideration of sex-specific characteristics.

## Figures and Tables

**Table 1:** General characteristics of the study participants according to sex and phase angle group.

Variables	Male				Female			
	Total	High PhA	Low PhA	*p*	Total	High PhA	Low PhA	*p*
Stroke Etiology (ischemic/ hemorrhagic)	65/54	27/17	38/37	0.258	74/57	33/27	41/30	0.752
Affected side (right/left /bilateral /quadriplegia)	48/61/1/9	23/20/0/1	25/41/1/8	0.107	61/60/0/11	34/24/0/4	27/36/0/7	0.073
Sarcopenia (Yes/No)	53/66	11/33	42/33	0.001 *	59/72	16/44	43/28	<0.001 *
Age (years)	65.09 ± 12.26	59.84 ± 11.39	68.08 ± 11.79	<0.001 *	68.46 ± 14.51	63.28 ± 14.15	72.83 ± 13.41	<0.001 *
Height (cm)	169.13 ± 7.20	170.11 ± 8.12	168.56 ± 6.60	0.258	157.66 ± 6.40	158.02 ± 7.40	157.35 ± 5.46	0.556
Weight (kg)	65.91± 12.70	70.12 ± 12.42	63.45 ± 12.30	0.005 *	57.32 ± 10.71	59.23 ± 9.15	55.71 ± 11.69	0.061
BMI (kg/m^2^)	22.86 ± 3.67	24.22 ± 3.25	22.07 ± 3.70	0.002 *	23.02 ± 4.26	23.65 ± 4.29	22.49 ± 4.20	0.123

* *p* < 0.05, PhA: Phase angle; BMI: Body mass index.

**Table 2:** Comparison of Functional Outcomes According to Phase Angle Groups.

Variables	Male			Female		
	High PhA	Low PhA	*p*	High PhA	Low PhA	*p*
BBS (Score)	27.11 ± 19.41	16.21 ± 17.33	0.002 *	21.80 ± 18.35	12.18 ± 15.73	0.002 *
FAC (Score)	1.82 ± 1.69	0.95 ± 1.34	0.002 *	1.28 ± 1.45	0.80 ± 1.20	0.040 *
K-MBI (Score)	48.50 ± 23.64	33.20 ± 22.64	0.001 *	42.88 ± 26.04	30.06 ± 21.04	0.002 *

* *p* < 0.05, PhA: Phase angle; BBS: Berg balance scale; FAC: Functional ambulation category; K-MBI: Korean version of the modified Barthel index.

**Table 3:** Sex-stratified logistic regression analysis of associations between functional variables and low phase angle.

Variables	Male				Female			
	Crude OR (95% CI)	*p*	aOR (95% CI)	*p*	Crude OR (95% CI)	*p*	aOR (95% CI)	*p*
BBS < 41	0.30 (0.12–0.73)	0.008 *	0.30 (0.12–0.77)	0.008 *	0.48 (0.19–1.20)	0.115	0.68 (0.10–4.60)	0.687
FAC < 3	0.27 (0.11–0.65)	0.008 *	0.27 (0.11–0.65)	0.005 *	0.58 (0.23–1.49)	0.258	2.12 (0.29–15.71)	0.454
K-MBI < 75	0.33 (0.08–1.41)	0.138	0.33 (0.08–1.33)	0.138	0.08 (0.01–0.66)	0.019 *	0.06 (0.01–0.71)	0.022 *

* *p* < 0.05, OR: Odds ratio; aOR: adjusted Odds ratio; CI: Confidence interval; BBS: Berg balance scale; FAC: Functional ambulation category; K-MBI: Korean version of the modified Barthel index.

## Data Availability

The data can be requested from the corresponding author and will be released on reasonable request.

## Abbreviations

The following abbreviations are used in this manuscript:

ADL	Activities of daily living
BIA	Bioelectrical impedance analysis
PhA	Phase angle
BBS	Berg balance scale
FAC	Functional ambulation category
K-MBI	Korean version of the modified Barthel index
OR	Odds ratio
aOR	Adjusted odds ratio
CI	Confidence interval
